# Increased Flight Initiation Distance (FID) in Golden Marmots (*Marmota caudata aurea*) Responding to Domestic Dogs in A Landscape of Human Disturbance

**DOI:** 10.3390/ani9090605

**Published:** 2019-08-26

**Authors:** Muhammad Zaman, Bryony A. Tolhurst, Mengyan Zhu, Guangshun Jiang

**Affiliations:** 1 Feline Research Center of Chinese State Forestry and Grassland Administration, College of Wildlife and Protected Areas, Northeast Forestry University, Harbin 150040, China; 2 Ecology, Conservation and Zoonosis Research and Enterprise Group, University of Brighton, Brighton BN2 4GJ, UK

**Keywords:** Flight Initiation Distance, disturbance, domestic dog, marmot, burrow substrate

## Abstract

**Simple Summary:**

Humans and domestic dogs may alarm wild animals, and the extent of this can be measured using Flight Initiation Distance (FID). Golden marmots are preyed on by globally-endangered predators such as the snow leopard, and are baited by humans with dogs, potentially causing FID to increase. We measured FID in 72 marmots from four colonies in the Karakoram range, Pakistan. Marmots were approached by a person on foot with a leashed dog, and by a person on their own to compare FID between the two. Additionally, we recorded background signs of human activity, namely roads, and presence of people other than the experimenters. We measured other aspects of the environment that might have affected marmot behavior such as marmot group size and age/sex, how visible each colony was, and colony substrate. The dog caused greater FID than the person alone, and adult marmots nearer to roads showed greater FID. However, marmot age and colony substrate had more marked impacts on FID, which was also greater at lower elevations where there were clusters of human settlements and livestock pasture. Further research should be conducted to explore some of these effects further and to find out whether increased FID affects marmot survival and breeding success.

**Abstract:**

Humans and dogs initiate measurable escape responses in wild animals including flight initiation distance (FID), with potentially negative consequences. Golden marmots are important prey for endangered carnivores and are subject to human persecution including via marmot baiting with dogs. We quantified FID at four marmot colonies (72 individuals) in the Karakoram range, Pakistan in response to approach by a pedestrian with a leashed dog versus approach by a pedestrian alone (i.e., a control). Additionally, we related FID to background variables of human activity, namely proximity to roads, and presence of other pedestrians in the vicinity of study sites during sampling. We also controlled for potential environmental and social covariates (e.g., group size, age and sex, and colony substrate). Dogs initiated greater FID than pedestrians alone, and there was evidence that roads increased FID. However, these effects were weaker than those of marmot age and colony substrate. FID was greater at lower elevations, but this may reflect the clustering in these zones of human settlements and livestock pasture. Further work is needed elucidate the importance of colony substrate (linked to ease of human persecution), the effect of settlements and pasture, and the impact of increased FID on marmot fitness.

## 1. Introduction

Prey perception of predation risk is a key driver of escape decisions [1]. One such decision: the distance from an approaching predator at which a prey animal initiates flight, reflects dynamic fitness trade-offs between fleeing and staying [2], and is termed Flight Initiation Distance (FID) [3]. FID is correlated with two additional influential parameters: starting distance (SD: distance between predator and prey when the former begins its approach); and alert distance (AD: predator–prey distance when prey responds overtly to the presence of the predator) [4]. As prey perceive humans as predators [4], the standard FID protocol involves measuring focal prey animal response to approach by a single pedestrian [1].

Different types of human stimulus such as noise from road traffic or other forms of mechanization, and disturbance from pedestrians and domestic dogs (*Canis familiaris*) have differential effects on prey animals [5,6]. Approach by pedestrians appears to initiate greater AD and FID than mechanized stimuli (e.g., [7]) but prey behavior can be substantially modified by background noise (e.g., traffic recordings paired with predator calls in playback experiments increased AD and FID in black-tailed prairie dogs (*Cynomys ludovicianus*) [8]). Free-ranging domestic dogs are associated with significant lethal and sub-lethal effects on prey animals [7] and it is therefore not surprising that many studies have shown greater FID in response to humans with dogs than humans alone (e.g., [9]). However, other authors report humans eliciting equal or greater FID than domestic dogs [10], or increased FID contingent on dogs being unleashed. Most studies on domestic dogs and wildlife FID have focused on responses on or off hiking trails and show a difference between the two (e.g., [7]). Responses also differ according to previous exposure ([11], individual personality [12], and hunting pressure [10]. Hunted populations are reported to display greater FIDs than non-hunted ones (e.g., giraffes and reindeer [9,13], and temporal changes in human presence elicit associated changes in FID [14]. Nonetheless, wild animal habituation to the presence of humans and related infrastructure is also common and widespread [15,16]. 

Multiple extrinsic (environmental) and intrinsic (social, morphological, and physiological) factors influence prey escape responses to human and non-human predators [3], the latter including body condition, body temperature [17,18], and degree of crypsis ([19]. In particular, the effect of group size on FID is well-documented but highly inconsistent between studies and includes strong positive [3] and strong negative [20,21], effects in terrestrial organisms. Biases in escape decisions relating to sex and reproductive status are again influential and arguably more consistent-generally involving greater FID and/or AD in females (e.g., Columbian black-tailed deer (*Odocoileus hemionus columbianus*) [22]) especially those with young (e.g., Alpine marmots (*M. marmota*) [23]). Finally, mammalian prey species are very sensitive to spatial variables in their environment [16], particularly those affecting visibility and detection probability such as topography and vegetation height. For example, brown hare (*Lepus europaeus*) mount earlier escape responses in shorter grass [24]. Additionally, for burrowing species, proximity to a refuge is an important predictor of FID (e.g., plateau pika (*Ochotona curzoniae*) [25]).

Marmots (*Marmota; Sciuridae*) are ideal model systems for investigating factors affecting FID and AD as their anti-predator behavior is well understood (e.g., [26,27]). Golden marmots (*Marmota caudata aurea*) are a cooperatively breeding burrowing species that inhabit high-altitude environments across Afghanistan, Pakistan, Kazakhstan, and China [28,29], and are important prey for golden eagle (*Aquila chrysaetos*), Tibetan wolf *(Canis lupus chancho)* and snow leopard *(Panthera uncia)* [29,30]. They are currently classified as *least concern* by the International Union for the Conservation of Nature (IUCN) however their population trend requires monitoring [30]. Unlike in other marmot species, golden marmot social and reproductive systems are not well known, although they are believed to be facultative biennial breeders due to the harsh unproductive environments they inhabit [31]. Their activity is restricted to four months during the Spring/Summer, when they must reproduce and accumulate sufficient fat reserves and body mass to survive over-winter hibernation under snow cover [26]. Asian marmot species are subject to significant hunting pressure throughout their ranges [32,33] and golden marmots are commonly baited with domestic dogs for entertainment and/or persecuted as pests (Zaman personal observation). Persecution is at least partially derived from the perception that marmots compete for overwinter forage with livestock animals, and with Himalayan ibex (*Capra siberica sakeen*), which is widely hunted in the region thus generating substantial income locally (Zaman *pers. obs*.).

We compared golden marmot FID in response to two different stimuli–approach by a pedestrian with a domestic dog on a long leash (the treatment), and approach by a pedestrian only (the control). We expected the dog to evoke greater marmot FID than a pedestrian alone, due to the local ubiquity of marmot baiting with dogs being likely to engender sensitization rather than habituation. We also determined the influence of background human activity on marmot FID in the form of proximity to roads, and the presence of additional pedestrians in view during sampling sessions (e.g., tourists, livestock farmers). These were also expected to negatively predict FID due to the high background level of anthropogenic disturbance and persecution. We controlled for the potentially confounding effects of group size, proximity to a refuge (i.e., burrow), age, sex, topography, vegetation height, and elevation.

## 2. Materials and Methods

### 2.1. Study Area

**Figure 1:**
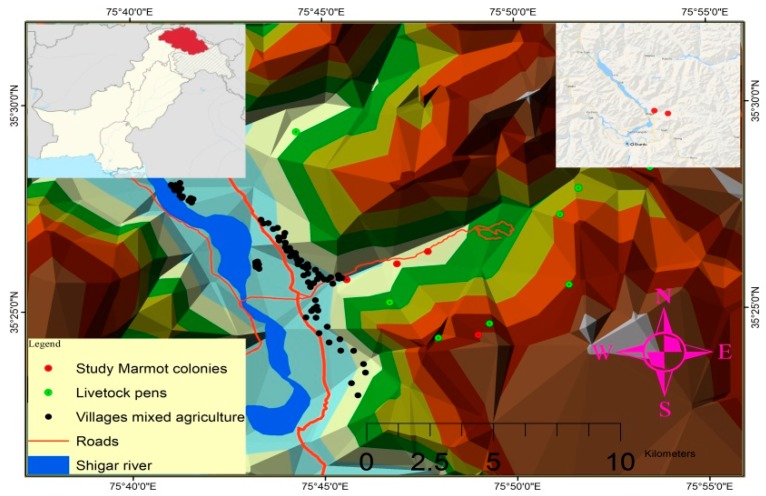
Study area in the Shigar Valley, Karakoram Range in North-East Pakistan, showing the location of the four marmot (*Marmota caudata aurea*) colonies in relation to roads, livestock pens, and villages, at different elevations.

The study was conducted in the Kanisker nullah area of the Shigar Valley within the Karakoram mountain range, located along the North bank of the river Indus in North-east Pakistan (Figure 1). It lies at 25°25′32″ N latitude and 75°42′59″ E longitude and covers an area of 4373 sq. km with an altitudinal range of 2260 to 8611 m above sea level including K2 (8611 m), Broad Peak (8047 m), Angel Peak (6858 m), and Skil Brum (7360 m) [34]. Annual precipitation in the area ranges from 200 mm to 600 mm, elevation from 2000 to 6000 m.a.s.l and snow depth from 5 to 12 inches [35]. Snow melt normally begins in mid April but can be later at higher elevations, and triggers increased marmot activity following emergence from hibernacula. Snow melt is significant because although marmots may use existing natural rocky crevices for parturition and rearing young, they also dig their own ‘earthen’ burrows, which requires the ground to be unfrozen and not under snow cover. Rocky burrows tend to be used for longer periods than earthen burrows because they are less affected by persecution from humans and by flooding. Four marmot colonies were selected for sampling comprising 5 to 20 individuals per colony, and divided equally between rocky and earthen burrow systems.

### 2.2. Live Capture and Marking of Marmots

A traditional live-trapping protocol was used to capture a sample of marmots at the four colonies, with assistance from experienced hunters and supervised by a veterinarian. The procedure involved closing multiple entrances to marmot earthen burrows by covering them with seabuck thorn twigs, followed by two-foot length nylon cloths (manufactured by Suzhou Jingang textile co., Ltd., Suzhou, China) to cease ventilation inside the burrows. A twelve feet long bamboo rod was then used to flush marmots out from inside the burrows and a 4-m long, 6-m wide net covered in ebony nylon apparel (Wenzhou Xinghai Fishing Tackle Co., Ltd., Wenzhou, China) was placed over the burrow system. When a marmot emerged, it became trapped in the net, and was subsequently placed inside a 5 cm-holed wooden box (50 cm × 50 cm × 50 cm) manufactured by local craftspeople. Animals were sexed using the criteria of observed mammary glands/descended testes (adult female/male respectively) and weighed to confirm age class. All animals were marked with permanent ink on the body with ebony colors [36]. Males were marked with two stripe marks on the body and females with one stripe; adults (marked on neck), yearling (back side), and juvenile (both neck and back). All marmots were immediately released at the site of capture following marking. Two marmots incurred very minor injuries potentially sustained by the trapping procedure and were therefore treated with antibiotic ointment and their condition assessed prior to release. All other marmots were completely unharmed. We undertook capture and handling under regulations for animal welfare and conservation under the Gilgit-Baltistan Wildlife Preservation Act 1975, and the North East Forestry University Guidelines for the Use of Animals in Research. 

### 2.3. Field Identification of Unmarked Individuals

Marmots in the study area were wary of humans and could not normally be observed easily at close-range. Capturing and marking a sample of animals therefore additionally provided a baseline against which to compare unmarked animals during subsequent field identification at mid-to-long range. This allowed adherence to the principles of the three Rs (National Centre for the Replacement, Refinement and Reduction in the Use of Animals in Research (NC3Rs) (https://www.nc3rs.org.uk/) via reduction of the number of captures required. Identification of age, sex, and reproductive status of unmarked animals was conducted (with reference to marked animals) on the basis of size, and prominent mammary glands in females/testes in males, observed during the bipedal vigilant stance.

### 2.4. Assessment of Flight Initiation Distance (FID) 

Field data were collected from the four colonies for four summer months over three years, from 2 May 2015 to 30 August 2017. Randomization was achieved using a balanced Latin square table generated for each year of the study where each of ‘pedestrian and dog approach’ (treatment) and ‘pedestrian only approach’ (control) were assigned to each colony four times per month. This allowed equal sampling across subjects and colonies whilst minimizing the influence of previous experience on marmot responses. Within this design, sampling sessions were additionally divided equally between morning and afternoon sessions to control for time of day effects. It was assumed that previous experience would minimally influence marmot behavior between study years. Each approach involved three investigators (one experimenter and two observers). All marmots included in analyses were either marked or otherwise identifiable to age and sex class, via observation through 10 × 42 binoculars. Additional colony-specific social and environmental variables, which reflected our hypotheses, were measured with the aid of a rangefinder or recorded retrospectively using Arc GIS 10.2 (see Table 1) [37]. All sessions were recorded using a video recorder, and any subsequent anomalies or missed measurements revisited and resolved. 

At the start of each sampling session the investigators arrived at a distance from the target colony that afforded a clear view without instigating marmot alarm behavior. A focal marmot was selected (usually the closest to the investigators), and its behavioral state recorded. The experimenter did not begin the approach until the focal marmot was in a relaxed state i.e., not sleeping but conducting any other non-vigilant activities such as foraging, auto-grooming or non-alarm communication [38,39]. This usually occurred within a few minutes of observation. The experimenter then walked towards the focal marmot with a domestic dog (*Canis familiaris*) on a lead, at a standardized speed of 0.5 to 1 m/s practiced prior to the field data collection [40,41]. The ‘pedestrian only approach’ (control) followed an identical protocol without the dog. Experimenters switched between sampling sessions but wore similar clothing [38,42,43,44]. The experimenter dropped a flag at each of the following: (i) the point of onset of the approach; (ii) experimenter position when the focal marmot responded to the approach (e.g., by moving its head in experimenter’s direction); and (iii) experimenter location when the marmot started to flee, usually by running into a burrow. Subsequently, the following were recorded using a standard tape measure: (i) starting distance SD (first flag to initial position); (ii) alert distance AD (second flag to initial position); and FID (third flag to initial position), all as straight-line measurements in meters [45]. 

### 2.5. Data Analyses 

Data were analyzed within a multiple regression GL(M)M null hypothesis significance testing (NHST) framework computed in R statistical software version 3.5.0 (www.r-project.org) (R Core Development Team 2018). The response variable (FID) was non-normal and over-dispersed and was subsequently modelled using a quasi-poisson distribution and a log link function. The response was subsequently regressed against 11 explanatory candidate fixed effects selected *a priori* in accordance with the hypotheses (Table 1). We initially ran two bivariate GLMs to test for an effect on FID of: (i) capture status (whether marmots were marked or not, measured as unmark/mark, 0,1); and (ii) year of study, on FID. Capture status was non-significant (coefficient = 0.05 ± 0.11; t = 0.43, *P* = 0.67) and was consequently excluded from further model selection. Putative differential marmot responses to proximity to roads and background human presence between age and sex classes were additionally investigated via the introduction of two-way interaction terms between: (i) Distance to roads and age/sex class; and (ii) human presence and age/sex class. Further, because observers noted that marmots appeared to tolerate a closer approach when near burrows in rocky colonies, an interaction term between burrow type and distance to refuge was included in the model. A backwards model selection procedure was then employed where all fixed main effects and interactions were initially entered together before sequential removal of non-significant variables at the 95% level, based on lowest deviance (*F*) values. We included colony ID as a random effect in the model, to account for repeat observations of the same colonies over time [45]. Initially, individual ID was additionally included as a random factor, but poor model fit (i.e., lack of convergence) resulted in removal of this variable. The random structure of the GL(M)M was computed using the glmmPQL command in package MASS. Quasipoisson models are not consistent with Akaike’s Information Criterion (AIC) comparisons hence comparison of best-fit models using information statistics was not conducted.

### 2.6. Ethical Approval.

In the study, the field data were collected following ethical review by Northeast Forestry University, Harbin, China, following their guidelines for the use of animals in research and approving PhD study “2016DFH425”. The authors complied with the wild animal protection law of the People’s Republic of China.

## 3. Results

Data were collected from a total of 72 individuals, including 37 males (13 adults, 10 yearlings, and 14 juveniles) and 35 females (19 adults, 9 yearlings, and 7 juveniles). Of these, 10 were marked (divided equally between age and sex classes). Marmots colonies were sampled along an elevational gradient of 2585 meters (i.e., ranging from form 2207-4792 m.a.s.l) and varying distance to roads (Table 2). Mean FID overall ± SD was greater for the pedestrian and dog treatment (18.43 ± 23.01) relative to the pedestrian alone (control) (11.46 ± 18.28). This difference was significant at the 99% level when controlling for other variables (Table 3). However, there were also strongly significant inter-annual and demographic effects, and two of these (age, sex, and burrow type) when combined, accounted for over 50% of the variance in FID explained by the final model (Table 3). Conversely, treatment (difference between pedestrians and dogs) accounted for only 4.38% of the variance. Neither human presence, slope nor the main effects of distance to roads and distance to refuge influenced FID (*F* (1, 628) always < 0.001, *P* always > 0.05). Adults fled at greater distances than both juveniles and yearlings except for when comparing adult males and yearling females; and marmots in earthen burrow systems fled at greater distances than in rocky ones (Table 3). There was a strongly significant interaction between proximity to roads and age/sex (*F* (4, 619) = 311.86, *P* < 0.001) that appeared to show a negative correlation for adults only (coefficient = −0.0002 ± 0.003, t = −3.079, *P* < 0.01). A strong negative elevation effect was observed where FID decreased with height above sea level (*F* (1, 629) = 766.00, *P* « 0.001); and with vegetation height where short vegetation was associated with greater FID (*F* (1, 627) = 960.31, *P* « 0.001). Data from 2016 exhibited a significant increase in FID relative to the other two years (Table 3). There was a significant interaction between distance to a refuge and burrow type, showing that the former positively predicted FID for rocky burrows only, although this relationship was relatively weak (*F* (1, 624) = 94.50, *P* < 0.05). Viewshed positively predicted FID, i.e., FID increased with increasing visibility, but again this was a weak effect (*F* (1, 628) = 960.31, *P* < 0.05). Colony ID accounted for only 0.02% of the total variance (1.62/7738) (Table 3).

## 4. Discussion

We found that Flight Initiation Distance (FID) in golden marmots was greater in response to approaching humans with dogs than to humans alone, which is consistent with our hypothesis. However, we detected little overall effect of background human presence as measured by our variables, with only a moderate negative impact of proximity to roads on FID to partially support our second hypothesis. Nonetheless, road traffic is a constant and relatively low amplitude sound, to which habituation is likely, and elevated FID costly. Conversely, sudden noises are powerful cues in triggering anti-predator behavior in herbivores [46,47]. The pronounced inter-annual effect we detected corresponds to elevated intensity of quarrying for boulders in the area close to the colonies in 2016 and gives credence to unpredictable and intermittent noises initiating more intense behavioral changes. Further, in retrospect we believe that the negative effect of elevation on flight response is likely to be an artefact, at least in part, of the concentration of both livestock pens and villages at lower elevations (see Figure 1). This merits further work exploring and accounting for intercorrelations between elevation and the configuration of human settlements and activity.

The impact of extrinsic and intrinsic covariates was broadly in keeping with the literature, especially the respective positive/negative associations between FID and visibility/vegetation height. We interpreted these findings as marmots being able to see an approaching predator more easily when visibility was greater, thereby mounting a flight response at an optimal distance, although this may be moderated by the tradeoff between predator detection and the concealment provided by cover (e.g., 16). As marmots exhibit cooperative anti-predator behavior, FID may be further enhanced by open habitats allowing better transmission of acoustic warning signals, i.e., alarm calls [48]. The difference in FID between burrow substrate types was notable (marmots in earthen burrows fled at greater distances), as was the negative relationship between FID and distance to a refuge being restricted to earthen burrows only. These effects may relate to the relative immunity of marmots in rocky burrow systems against human persecution–and merits further investigation. The lack of a relationship between FID and group size was unexpected given the propensity of other studies that show strong (albeit opposing) patterns (e.g., [41]). It is possible that our threshold distance of 10 m to delineate a group was not sensitive enough to detect an effect in either direction. An additional potential limitation of the study is reduced power arising from repeat observations of individuals over time (i.e., pseudo-replication), which we were unable to explicitly model due to computational difficulties. However, this effect is likely to have been minimal due to the comparatively large numbers of individuals sampled.

The findings concerning demographic effects are consistent with predictions that adults (especially females) are likely to be most wary, reinforcing the need to control for marmot age and sex when measuring responses to anthropogenic disturbance. We were not able to investigate the impact of stage of reproductive cycle on sex-specific biases in FID due to the lack of opportunities for comparative studies in the winter when marmots are largely inactive under snow cover. However, motivational state driven by reproductive status can be important in fitness tradeoffs governing the distance at which prey choose to flee, with early-onset flight being offset by the calorific gain of foraging or the cost of lost mating opportunities [49]. For example female Apennine chamois (*Rupicapra pyrenaica ornate*) fled earlier than males when offspring were present [50] and female Columbian black-tailed deer (*Odocoileus hemionus columbianus*) fled at greater distances from approach by an armed pedestrian relative to males, but only during the breeding season [3]. As golden marmots are likely income breeders [26], it is reasonable to surmise that elevated FID in response to background human presence and persecution incurs a cost, which may be asymmetrical between sexes. Currently there is limited longitudinal research linking human activity (either in itself or as a proxy for predation pressure) to reduced fitness in prey, although negative long-term effects have been demonstrated in some species with short generation times e.g., increased mortality in Ambon damselfish (*Pomacentrus amboinensis*) treated to noise [51]. It is not known whether increased FID in response to various stimuli in the current study was ultimately associated with increased mortality or reduced birth rates. Certainly, further work is needed to determine the impact of elevated FID on life history parameters (mortality, fitness) and the mechanisms that determine them in this system. There is also growing evidence to suggest that an animal may not always exhibit marked behavioral change even though it is disturbed and potentially highly stressed, because such behaviors would be too costly [4,5]. Therefore, physiological indicators of stress in marmots that are independent of FID (and other measures of anti-predator behaviour) should be measured alongside behavioural correlates of anthropogenic disturbance in further investigations. 

## 5. Conclusions

Overall, we surmised that both immediate encounters with dogs, and background indicators of broader human activity were important drivers of marmot behavior, and that in general marmots were sensitized rather than habituated to human disturbance. However, further work is recommended focusing on the apparent link between human disturbance and burrow (colony) substrate; and the spatial relationships between elevation and human settlement. 

## Figures and Tables

**Table 1:** List and description of all candidate fixed main effects for predicting Flight Initiation Distance (FID) in golden marmots. * not possible to determine sex in juveniles; TIN = triangulated irregular network; DEM (50 m) = digital elevation model at the scale of 50 m.

Variable Name	Description	Factor Levels
Treatment	Focal marmot approach by humans/dog	Pedestrian only
		Pedestrian and dog
Human presence	Presence of additional pedestrians within view (e.g., tourist hikers, livestock farmers)	Binary—humans present (1)
		Humans absent (0)
Agesex *	Combined age and sex class of focal marmot	Adult female
		Adult male
		Juvenile *
		Yearling female Yearling male
Distance to road	Distance (m) from center of colony to nearest road measured using Arc GIS (10.2) near analysis tool	Continuous
Group size	Number of conspecifics within 10 m of focal marmot	Continuous
Distance to refuge	Distance (m) from focal marmot’s initial position to the nearest burrow	Continuous
Burrow type	Comprised of earthen or rocky type burrows	Earthen
		Rocky
Vegetation height	Grass/shrub height estimated by eye	Tall (>30 cm)
		Short (0–30 cm)
Viewshed	A measure of visibility on a TIN from the center of each colony to several observation points. Higher values represent greater visibility (i.e., of colony to predators).	Continuous
Slope	Slope of colony derived from a DEM (50 m) within the 3D Analyst extension of Arc GIS (10.2)	Continuous
Elevation (m.a.s.l)	Elevation in meters above sea level	Continuous
Year	Year of study	2015
	(4-month study period per year)	2016
		2017
Starting Distance (SD)	Distance (m) between experimenter and focal marmot when the former begins approach	Continuous

**Table 2:** Summary statistics for significant continuous variables (main effects and interactions) remaining in the final model predicting flight initiation distance (FID) in golden marmot (*Marmota caudata aurea*) colonies.

Variable	Mean (± SE)	Minimum Value	Maximum Value
Elevation (m.a.s.l)	2858.10 ± 29.99	2207	4792
Viewshed (°)	29.32 ± 14.82	20.12	50.88
Distance to refuge (m)	1.03 ± 4.54	0	221
Distance to roads (m)	811.63 ± 44.4	23.3	7987.5

**Table 3:** Parameter estimates from Maximum Likelihood for significant main effects predicting golden marmot FID within a Generalized Linear (Mixed) Model (GL(M)M) framework. Positive values show greater FID relative to the reference level, which is specified in each case. GLM variables are fixed main effects and GLMM variance components are random effects. Percentage deviance values are for each variable overall, as a proportion of the total deviance explained by the model (residual deviance/null deviance = 59.08%). Significance levels are denoted as: * *P* < 0.05; ** *P* < 0.01; *** *P* < 0.001. Age/sex abbreviations are: *AF* = Adult Female; *AM* = Adult Male; *J* = Juvenile; *YM* = Yearling Male; *YF* = Yearling Female.

GLM			GLMM		
			Variance Component	Variance	% of Total Variance
			Colony ID	0.162	0.02
			Residual	3.731	0.04
Explanatory variable (post hoc pairwise comparisons)	Estimate	Std. Error	% deviance explained *	*t*	*P*
Treatment (Dog versus pedestrian)	0.527	0.186	4.380	2.823	*
Vegetation height (Long versus short)	0.660	0.100	6.360	−6.563	**
Burrow type (Earthen versus rocky)	0.386	0.120	16.040	3.269	**
Age/sex class			35.470		
*AF* vs. *J*	−0.014	0.338	Na	−4.110	***
*AM* vs. *J*	−0.917	0.241	Na	3.807	***
*AF* vs. *YM*	−2.273	0.578	Na	−4.725	**
*AM* vs. *YF*	2.729	0.578	Na	4.725	***
*AM* vs. *YM*	−1.481	0.578	Na	−4.725	***
Year			8.500		
*2015* vs. *2016*	−0.409	0.151	Na	−2.703	**
*2017* vs. *2016*	−0.622	0.203	Na	−3.060	**
